# The Intrinsically Disordered Region of HBx and Virus–Host Interactions: Uncovering New Therapeutic Approaches for HBV and Cancer

**DOI:** 10.3390/ijms26083552

**Published:** 2025-04-10

**Authors:** Rodrigo A. Villanueva, Alejandra Loyola

**Affiliations:** 1 Centro Científico y Tecnológico de Excelencia Ciencia & Vida, Fundación Ciencia & Vida, Santiago 8580702, Chile; 2 Facultad de Ciencias, Universidad San Sebastián, Santiago 7510602, Chile

**Keywords:** hepatitis B virus (HBV), HBV X protein, HBx protein, intrinsically disordered region (IDR), viral regulatory protein, cccDNA, viral mini-chromosome regulation, antiviral target

## Abstract

Human viral infections remain a significant global health challenge, contributing to a substantial number of cancer cases worldwide. Among them, infections with oncoviruses such as hepatitis B virus (HBV) and hepatitis C virus (HCV) are key drivers of hepatocellular carcinoma (HCC). Despite the availability of an effective HBV vaccine since the 1980s, millions remain chronically infected due to the persistence of covalently closed circular DNA (cccDNA) as a reservoir in hepatocytes. Current antiviral therapies, including nucleos(t)ide analogs and interferon, effectively suppress viral replication but fail to eliminate cccDNA, underscoring the urgent need for innovative therapeutic strategies. Direct-acting antiviral agents (DAAs), which have revolutionized HCV treatment with high cure rates, offer a promising model for HBV therapy. A particularly attractive target is the intrinsically disordered region (IDR) of the HBx protein, which regulates cccDNA transcription, viral replication, and oncogenesis by interacting with key host proteins. DAAs targeting these interactions could inhibit viral persistence, suppress oncogenic signaling, and overcome treatment resistance. This review highlights the potential of HBx-directed DAAs to complement existing therapies, offering renewed hope for a functional HBV cure and reduced cancer risk.

## 1. Human Viral Infections and Cancer

The diseases caused by human viruses present important challenges to global health. Over the years, main advancements, such as vaccination campaigns, have been organized to manage several diseases. For instance, the World Health Organization (WHO) put together a worldwide effort to eradicate smallpox. The campaign was officially initiated in 1967, and it was led by Donald Henderson. The WHO officially declared smallpox to be globally eradicated by the 1980s [1,2]. Despite these achievements, several viral infections remain without effective vaccines, requiring continuous treatment for the infected individuals. A substantial number of viruses have also been linked to cancer development in humans, contributing to more than 12 million new cancer diagnoses and nearly 8 million fatalities globally in 2008 [3,4,5]. Pathogens, including viruses, bacteria, and parasites, account for about one-sixth of all global cancer cases [5,6]. Among these, oncoviruses contribute to 12% of all cancer cases, rendering over 1.5 million newly diagnosed cases annually. The group of human oncoviruses includes seven members: human papillomavirus (HPV), human herpesvirus 8 (Kaposi’s sarcoma-associated herpesvirus, KSHV), human T-cell lymphotropic virus 1 (HTLV-1), Merkel cell polyomavirus (MCV), Epstein–Barr virus (EBV), and the hepatitis B and C viruses (HBV and HCV) [4]. Hepatitis B virus (HBV) is a DNA virus associated with hepatocellular carcinoma (HCC), promoting liver cancer via persistent infection and chronic inflammation [7]. Oncoviruses trigger cancer development through multiple mechanisms, including the direct impact of viral proteins on cellular proliferation and survival, along with indirect effects via sustained inflammation and immune evasion, as observed in HBV infections.

## 2. HBV Infection: A Global Health Concern

HBV infection is a major global health concern, affecting millions of individuals across the world. Although the introduction of an effective preventive vaccine in the 1980s significantly diminished the rate of new cases, a therapeutic cure for chronic HBV infection remains elusive [8]. Acute infections can develop into chronic HBV (in 5 to 10% of infected adults), resulting in severe liver complications, including cirrhosis, liver failure, and HCC [4,8]. According to the WHO, around 257 million individuals were chronically infected in 2015, constituting 3.5% of the human population [9]. Thus, chronic HBV remains a serious public health challenge globally [10].

The HBV virion, belonging to the *Hepadnaviridae* family, is a small (approximately 45 nm diameter), enveloped virus particle with a DNA genome. The virus spreads through parenteral, sexual, and mother-to-child transmission routes, often resulting in persistent infections. HBV is known to infect a limited host range, primarily targeting hepatocytes in humans and some animal species, including woodchucks and ground squirrels [11]. The HBV genome within the virion consists of a partially double-stranded, circular DNA molecule of 3.2 kb, known as relaxed circular DNA (rcDNA) [11]. Upon cell entry, host enzymes convert this rcDNA into covalently closed circular DNA (cccDNA) in the nucleus, facilitating long-term infection [12].

HBV is classified into four serological subtypes (adr, adw, ayr, and ayw), determined based on variations in the HBsAg HBV surface antigens. It is also classified into ten genotypes (A to J) based on sequence divergence of >8% in the DNA genome, which are linked to distinct geographic distributions [13]. Genotype D is globally distributed, while genotype A is predominantly found in Northwest Europe, North America, and Africa; genotypes B and C are widespread in Asia, whereas genotypes F and H are native to Latin America, with genotype F also present in Alaska, and the F1b subgenotype is particularly prevalent in Chile [14,15,16]. Genetic diversity among the HBV genotypes is also linked to varying disease outcomes. For example, genotype F is associated with rapid progression to liver cancer, while genotype H, common in Mexico, is associated with a lower prevalence of liver cancer and generally milder outcomes [14].

## 3. HBV Genome and Replication Cycle

The HBV genome encodes four primary proteins—the core, polymerase (P), surface proteins, and the X protein (HBx)—with overlapping open reading frames. The expression of these viral proteins is regulated at both the transcriptional and post-transcriptional levels [17]. HBV replicates its genome via reverse transcription. The infection cycle initiates when the virion attaches first to heparan sulfate proteoglycans (HSPGs) and then to the sodium taurocholate co-transporting polypeptide (NTCP), both at the surface of hepatocytes, triggering internalization by endocytosis [18]. After entering the cell, the viral nucleocapsid is released into the cytoplasm and then transported toward the nucleus, where the nuclear pore complex (NPC) recognizes the nucleocapsid, allowing only partially disassembled capsids to pass through. The viral rcDNA is then released into the nucleus, where it is repaired and converted into cccDNA by the nuclear enzymatic machinery, forming an episomal mini-chromosome responsible for viral persistence [19]. This nuclear cccDNA serves as a template for all viral transcripts, primarily synthesized by RNA polymerase II [17,19]. Among the viral transcripts, a 3.4 kb pre-genomic RNA (pgRNA) is both longer and co-linear with the cccDNA template, representing the full transcript of the viral genome. The pgRNA encodes both the viral polymerase and core reading frames. Once synthesized, the pgRNA is translated, and both the viral core (hyper-phosphorylated) and P proteins assemble into immature capsids, where reverse transcription of pgRNA into rcDNA occurs [20]. Stable mature capsids containing hypo- or de-phosphorylated capsid proteins either re-enter the nucleus to maintain the cccDNA reservoir or are enveloped by viral surface proteins within the endoplasmic reticulum and released as mature virions via the multivesicular body pathway [21]. Additionally, linear HBV DNA may be generated as a byproduct of reverse transcription and can either integrate into the host genome or convert to cccDNA [22]. Integration events often promote HCC through chromosomal instability and mutagenesis. While integrated HBV DNA does not support full viral replication, it still drives surface protein and mRNA production [23]. Recent studies have also detected HBV DNA integrated into mitochondrial genomes, but the functional implications of this observation are still under investigation [24]. Further laboratory research is essential to elucidate the role of mitochondrial networks in HBV replication.

## 4. The Puzzle of Sequences, Partial Structures, and Different Functions of the HBV Canonical HBx

**Figure 1:**
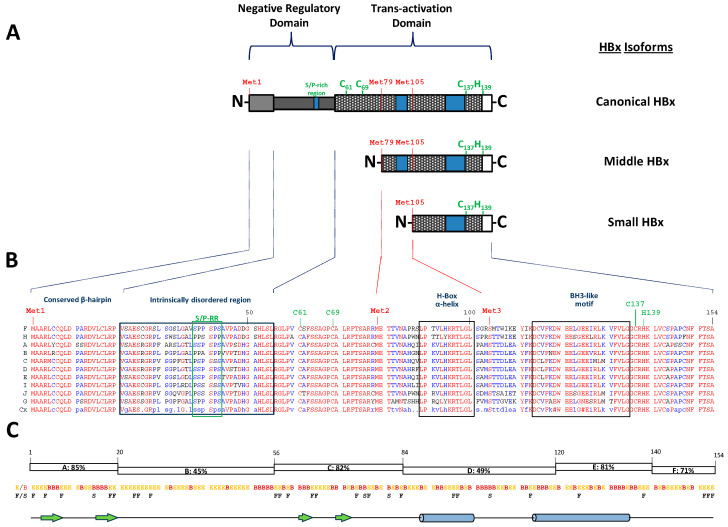
The HBV HBx protein. (**A**) A schematic representation of the canonical HBx protein and its domain organization. HBx consists of an N-terminal negative regulatory domain and a C-terminal transactivation domain, as indicated at the top. Below, two N-terminally truncated HBx isoforms (middle and small) are shown, highlighting their divergence from the canonical HBx. (**B**) Multiple sequence alignment of HBx from representative HBV genotypes. The first column denotes the genotype, while the last lane (Cx) represents the consensus sequence. Residues are color-coded to indicate their conservation: highly conserved (red), similar but not identical (blue), or variable or less conserved (black). Dots indicate that further criteria are needed to determine the conserved residues. Several key structural and functional sequence blocks are highlighted, including the conserved β-hairpin, the intrinsically disordered region (IDR), the H-box α-helix, and the BH3-like motif. Additionally, the positions of conserved methionine residues within the HBx open reading frame and the Cys and His residues forming the proposed CCCH-type zinc finger motif are indicated. (**C**) The top panel shows segment-wise conservation of HBx, represented by open blocks spanning the extent of each segment. Below, a Consurf analysis displays the residue exposure status (exposed (E) or buried (B)). Further down, the Consurf analysis categorizes each residue as functional (F) or structural (S). Finally, the predicted secondary structure of HBx is shown at the bottom.

HBx, the smallest protein encoded by the hepatitis B virus genome, is produced from a gene comprising 465 nucleotides and encodes a polypeptide of 154 amino acids with a molecular mass of 16.6 kDa [11] (Figure 1A).

The designation “HBV protein X” originated due to the absence of any similar protein in either host organisms or other viruses. Even at the time of its initial identification in the 1980s, its specific biological role remained largely unclear [25,26]. HBx is the only known regulatory protein of HBV, transcribed from a small mRNA (~0.7 kb) under the regulation of the X promoter [17,27,28,29]. Its expression, however, is tightly controlled at both the pre- and post-transcriptional stages. Notably, the HBx gene contains two internal, evolutionarily conserved in-frame initiation codons, Met79 and Met105, leading to the production of truncated HBx isoforms—a middle-sized isoform (76 amino acids, 8.6 kDa) and a small isoform (51 residues, 5.8 kDa)—through alternative translation initiation [17,30,31,32] (Figure 1A). An additional mechanism involving an intragenic promoter between Met1 and Met79 also contributes to the synthesis of these smaller isoforms by modulating pre-transcriptional regulation [17,33]. Despite having divergent N-terminal domains, the canonical HBx and its isoforms share a common C-terminal region (Figure 1A). The N-terminal ends of these isoforms contain intrinsically disordered regions (IDRs), as previously analyzed [17]. Functionally, the full-length HBx and its truncated variants display differential roles in viral replication and subcellular localization. Historically, the significance of HBx isoforms has been overlooked, despite their detection via both conventional and the most advanced molecular biology techniques [17]. This gap highlights the potential for further groundbreaking discoveries in this field, although this review will predominantly focus on the canonical HBx protein.

HBx plays a pivotal role in HBV replication, cellular infection, and disease progression, though its precise mechanisms remain enigmatic [17,26,34,35,36]. The HBx protein is conserved across all mammalian *Hepadnaviridae* family members, such as woodchuck hepatitis B virus (WHBV), but is notably absent in avian hepadnaviruses like DHBV (duck hepatitis B virus). Disrupting HBx expression in the viral genome significantly impairs—although not completely—replication, though this can be restored by providing the protein in *trans* [37,38,39,40,41,42,43,44,45].

The primary amino acid sequence of human HBx is divided into six segments (A to F) based on their degree of conservation, as indicated by color in the amino acid sequence alignment (Figure 1B), which includes representative genomes of each HBV genotype [17,46]. Segment A (residues 1–20) is the most conserved (85%), followed by segments C (residues 56–84, 82%) and E (residues 120–140, 81%). These conserved regions are separated by less conserved domains—B (residues 21–55, 45%), D (residues 84–120, 49%), and F (residues 140–154, 71%) [17,46], as shown in Figure 1C. The canonical HBx protein is divided into two main functional regions [46,47,48] (Figure 1A, top). Its N-terminal domain (or negative regulatory domain, residues 1–50) includes a transrepressor activity region (residues 21–50) and a Ser/Pro-rich sequence (S/P-RR) [49]. This domain corresponds to an intrinsically disordered region (IDR) and encompasses segment B (residues 21–55) [17,50]. Some studies suggest that residues 1–42 within the N-terminal domain are dispensable for viral replication but may be implicated in hepatocarcinogenesis [51]. The IDR within the N-terminal domain, through interactions with host factors, significantly contributes to the protein’s role in promoting cellular pathogenesis. Proteins such as 14-3-3ζ, Pin1, and cortactin are known as binding partners of this region and are believed to play roles in pathogenesis and cellular transformation. Conversely, the C-terminal domain (or transactivation domain, residues 52–142) harbors a critical transactivation region essential for transcriptional activation and efficient HBV replication [47,52,53]. This domain is further subdivided into regions with distinct functions: residues 58–119 mediate signal transduction [54], residues 120–140 regulate nuclear transactivation [52,55], and the final 20 residues ensure protein stability [56]. Additionally, this domain contains mitochondrial localization sequences [57,58], suggesting a potential role in mitochondrial dynamics during HBV infection.

Despite considerable efforts to express and purify recombinant HBx for structural studies, its three-dimensional structure remains unresolved due to its poor solubility, low expression, and inherent instability [59,60]. Nonetheless, key structural features have been identified through in vitro analyses of peptide interactions, among other assays. The N-terminal A region adopts a β-hairpin structure (residues 2–21) that interacts with Spindlin1, a chromatin epigenetic reader involved in Wnt signaling modulation via protein arginine methyltransferase 2 (PRMT2) [61] (Figure 1C). In contrast, the intrinsically disordered B region contains a conserved S/P-RR motif (residues 38–43), implicated in various intracellular signaling pathways (Figure 1A–C). Notably, Ser31 and Ser41 within this IDR play regulatory roles in the structure, function, and pathogenic activities of HBx [62,63,64]. Binding sites for key host factors, including the 14-3-3ζ and cortactin proteins, are also mapped within this IDR. On the other hand, the C-terminal transactivation domain of HBx is relatively structured and includes a proposed CCCH-type zinc finger motif (Cys residues 61, 69, and 137 and His139) [60], as shown in Figure 1A. Additionally, this region encompasses the H-box (residues 88–100), an amphipathic α-helix critical for stability and protein interactions [65,66], and a BCL2 homology domain 3 (BH3) motif (BH3-like motif, residues 113–135) involved in apoptotic regulation [67,68] (Figure 1A–C).

The subcellular distribution of the HBx protein has long been debated. It is predominantly cytoplasmic, although nuclear localization has also been reported [59,69,70,71]. This variation is believed to depend on the expression level of HBx: higher protein abundance leads to cytoplasmic localization, while lower levels promote nuclear confinement [72,73]. Within the cytoplasm, HBx modulates mitochondrial metabolism, apoptosis pathways, and signal transduction cascades [38,74]. In the nucleus, it enhances the transcription of both viral and cellular genes by interacting with nuclear factors [75]. Although it does not directly bind double-stranded DNA [76], it binds single-stranded DNA, and it influences transcription by interacting with various proteins, including transcription factors and components of the nuclear machinery [77]. Furthermore, HBx has been implicated in the regulation of DNA repair pathways [78].

The interaction of HBx with host proteins significantly influences its multifunctional roles. For example, it facilitates HBV cccDNA transcription by targeting the DNA damage-binding protein 1 (DDB1)-containing E3 ubiquitin ligase complex, leading to the degradation of the structural maintenance of chromosomes 5/6 complex (Smc5/6 complex), an HBV host restriction factor [79]. On the other hand, its BH3-like motif mediates interactions with anti-apoptotic proteins like Bcl-2 and Bcl-xL, potentially contributing to HBV spread by inducing apoptosis in infected cells [80]. Additionally, HBx inhibits the tumor suppressor p53, disrupting p53-dependent apoptosis and DNA repair, which may promote cell survival and tumorigenesis [26]. The functional properties of HBx can vary depending on the HBV genotype, influencing both liver cancer progression and treatment responses. Notably, genotype-specific differences in HBx activity affect critical cellular pathways such as cell cycle regulation, apoptosis, and maintenance of cccDNA. For example, HBx from genotype C has been linked to a higher oncogenic potential, in part due to its stronger modulation of tumor suppressor pathways like p53. Additionally, HBx sequence variations can alter immune evasion mechanisms, potentially impacting how patients respond to antiviral therapies. These observations underscore the need to consider HBx genotypic diversity when developing targeted interventions for HBV-associated liver disease.

Furthermore, HBx contributes substantially to HBV immune evasion and the development of immune tolerance, especially in chronic infection and liver cancer. It disrupts antiviral signaling by interfering with pathways such as NF-κB and IRF-3, weakening interferon responses and reducing inflammatory cytokine production. HBx also downregulates MHC molecule expression, hindering immune recognition of infected hepatocytes. In hepatocellular carcinoma, it fosters an immunosuppressive environment by promoting cytokines like TGF-β and IL-10. These actions collectively support viral persistence and tumor progression, underscoring the importance of targeting HBx-driven immune modulation in future therapeutic approaches.

## 5. Epigenetic Regulation of HBV Transcription: Role of HBx Regarding cccDNA

**Figure 2:**
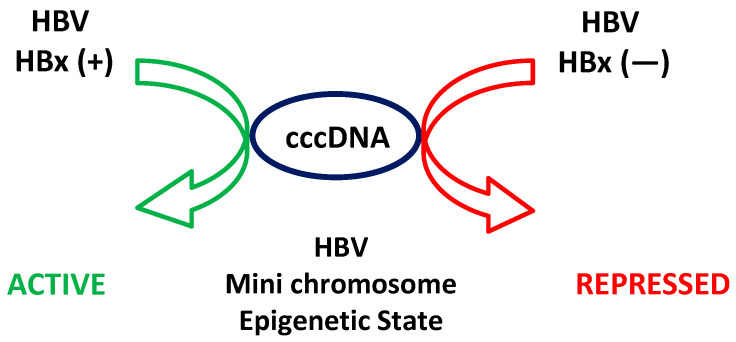
The current model of epigenetic regulation of cccDNA by HBx. The schematic figure illustrates the epigenetic control of HBV’s covalently closed circular DNA (cccDNA) by HBx. (**Left panel**): In the presence of wild-type (WT) HBV, where canonical HBx is expressed, the HBV mini-chromosome adopts an active epigenetic state (green arrow), promoting transcription and viral replication. (**Right panel**): In contrast, when HBV variants lack canonical HBx expression, the HBV mini-chromosome remains in a repressed/inactive epigenetic state (red arrow), preventing transcription and viral replication.

Upon HBV infection, as indicated, the viral DNA is converted into a nuclear cccDNA, which serves as the template for all viral transcription. This cccDNA forms a mini-chromosome in the nucleus, organized with regularly spaced nucleosomes composed of both histone and non-histone proteins [81,82]. While both the HBV core and HBx proteins can bind to cccDNA and modify its structure, the core protein is not essential for transcriptional regulation [83]. HBx, however, plays a crucial role in regulating the transcription of cccDNA through multiple mechanisms, including the recruitment of transcriptional activators, the modulation of histone modifications, and interactions with host chromatin remodeling complexes [43,84,85]. HBx significantly influences the epigenetic landscape of cccDNA by altering histone modifications [86]. Research, including studies from our lab, indicates that histone post-translational modifications associated with cccDNA regulate viral transcription. Active transcription correlates with the methylation of histone H3 lysine 4 and hyperacetylation of histones, whereas repressed transcription is associated with the methylation of histone H4 on arginine 3, methylation of histone H3 on lysines 9 and 27, and hypoacetylation of histones [43,87,88,89]. Histone variants also impact HBV transcription, as demonstrated by histone H3.3, which assembles into cccDNA and activates transcription [90,91]. HBx interacts with various histone-modifying enzymes to promote a chromatin state favorable to transcription. It recruits histone acetyl transferases (HATs) such as p300, CREB-binding protein (CBP), and PCAF, enhancing histone acetylation and cccDNA transcriptional activation. Additionally, HBx interacts with histone deacetylases (HDACs) like HDAC1 and HDAC2 to dynamically regulate the histone acetylation status [43,92]. HBx also negatively modulates histone methyltransferases (HMTs) such as SUV39H1 and SETDB1, fostering a more transcriptionally active chromatin state [89]. Histone demethylases, including JMJD3 (KDM6B) and LSD1 (KDM1A), are modulated by HBx to promote transcriptional activation [88]. Furthermore, HBx interacts with other chromatin-associated proteins, such as the SWI/SNF chromatin remodeling complex, to alter nucleosome positioning and enhance transcriptional machinery accessibility [93]. It also interacts with protein arginine methyltransferase 5 (PRMT5), which symmetrically dimethylates arginine residues on histones, influencing the chromatin structure [94]. In line with all of this, the HBV HBx protein plays a fundamental role in modulating the epigenetic state of the cccDNA intermediate. The current working model of cccDNA metabolism indicates that, in the presence of HBx, cccDNA adopts an active chromatin state, enhancing HBV gene expression and viral progeny production. Conversely, in the absence of HBx (with an HBV (−) HBx mutant), cccDNA remains in an inactive chromatin state, like the low transcriptional activity observed in occult infections, as depicted in Figure 2. These infections, characterized by the persistence of viral DNA, reflect an inactive chromatin state of HBV cccDNA.

Thus, HBx is a multifunctional regulatory protein essential for HBV replication, viral persistence, and oncogenesis. It interacts with various host proteins and modulates the epigenetic regulation of cccDNA by altering histone modifications and the chromatin structure, thereby promoting viral gene expression. Despite its critical role, the precise mechanisms of HBx remain partially understood, making it a key target for future antiviral therapies.

## 6. The Role of Intrinsically Disordered Proteins (IDPs) and Regions (IDRs) in Cellular and Viral Interaction Networks

Recent studies underline that a large portion of proteins encoded by genomes across various organisms lack a fixed three-dimensional structure, yet they are central to many cellular processes. These proteins, known as intrinsically disordered proteins (IDPs) or intrinsically disordered regions (IDRs) (when the disorder is confined to specific sections) disregard the classical structure–function paradigm. Computational studies have revealed that more than one-third of eukaryotic proteins harbor long disordered regions, exceeding 30 amino acids [95,96]. IDPs are recognized for their ability to engage multiple partners, serving as dynamic hubs within cellular interaction networks critical to maintaining functional and structural integrity [97,98]. Their structural flexibility allows them to shift between distinct conformations depending on the interaction partner or remain disordered even when bound. Combined with post-translational modifications, this flexibility enables IDPs to fine-tune complex signaling pathways. The loss of key disordered hub proteins often results in severe, even lethal, outcomes. Examples of these kinds of proteins include cell cycle inhibitors p21 and p27 [99], tumor suppressor p53 [100], DNA repair proteins like BRCA1 (breast cancer 1) [101] and XPA [102], the neuroprotein α-synuclein [103], estrogen receptors (ERs), and the HBV X protein (HBx) [104] (Figure 1). These IDRs act as flexible linkers between structured domains or provide adaptable binding sites that become ordered upon interaction. By utilizing free energy derived from these interactions, IDPs drive essential, reversible interactions that are pivotal for cellular signaling [102,105]. Consequently, IDPs often form complexes with unique, adaptable architectures.

## 7. Disordered Regions Span Life Forms, Including Viruses

IDRs are found in proteins across bacteria, archaea, eukaryotes, and viruses. The structural freedom of these regions facilitates the evolution of novel interaction motifs that viruses exploit to manipulate host pathways. Viral disordered regions enhance adaptability by allowing high mutation rates, immune evasion, and multifunctional interactions targeting various host defense mechanisms [106,107]. Viral proteins often possess short disordered segments with few hydrophobic residues but high levels of polar amino acids, enabling them to form stable, hydrogen-bonded interactions without losing flexibility [108,109]. The disordered content in viral proteomes varies with the genome size: smaller viruses tend to have higher disorder (often over 50%), while larger viral genomes generally contain 20–40% disordered residues. For example, the avian carcinoma virus MH2 exhibits a high number of disordered residues, whereas the human coronavirus NL63 (HCoV-NL63) contains only a small percentage [108,109]. Human viruses such as HCV, HIV-1, and papillomaviruses have various disordered proteins, enhancing their ability to interact with numerous host factors [110].

The HCV non-structural phosphoprotein NS5A, critical for genome replication and anchored to membranes, features highly disordered regions (D2 and D3) at its C-terminus, serving as an interaction hub that disrupts host signaling and apoptotic processes [111]. Similarly, the human immunodeficiency virus 1 (HIV-1) Tat protein, a key regulator of viral transcription, and the Rev protein, involved in viral mRNA transport, display classic IDR characteristics like a high charge and low hydrophobicity, which are essential for their functional roles [108]. In the case of hepatitis delta virus (HDV), a satellite virus, the small δ-antigen (δAg) protein contains disordered domains that are crucial for its activity. Computational and experimental analyses of δAg across eight HDV groups have consistently shown significant levels of disorder [110,112,113].

## 8. Disordered Proteins and Disease: Opportunities for Therapeutic Targeting

Dysfunctional IDPs or proteins containing IDRs are frequently linked to various pathologies, including cancer, neurodegeneration, and viral infections, making them promising targets for drug discovery [114]. Since viral proteins often depend on IDRs for host manipulation, targeting these regions offers therapeutic potential [108,115]. However, their dynamic nature, lack of fixed binding sites, and transient interactions present unique challenges. Exploiting their tendency to undergo disorder-to-order transitions upon binding could pave the way for stabilizing interactions or designing inhibitors to disrupt critical pathways [110,115]. Developing effective therapeutic agents requires overcoming the structural variability of IDRs through approaches like the use of small molecules or peptides capable of stabilizing the bound conformations. Addressing these challenges could unlock new strategies for treating IDP-driven diseases and viral infections [116,117].

## 9. Protein–Protein Interactions Involving the Intrinsically Disordered Region of HBx

As already described, the HBV HBx protein is a multifunctional regulator crucial for the virus’s transcription, replication, and pathogenicity [35]. Through interactions with a wide array of host proteins, HBx modulates key cellular mechanisms such as transcription regulation, DNA repair, apoptosis, and signal transduction, among several other processes [35]. More than 150 host proteins have been identified as binding partners of HBx, underscoring the protein’s intricate involvement in host–pathogen dynamics and its role in viral persistence and immune evasion [17,104].

Among the different regions of HBx involved in host interactions, the IDR, located within residues 21 to 55 of its N-terminal domain (Figure 1A–C), is particularly important [17,35]. The structural plasticity of the IDR enables HBx to establish multiple dynamic interactions with cellular partners, serving as a key hub for the regulation of various processes essential to HBV replication and its associated oncogenesis [35]. Again, this adaptability of the IDR makes it an attractive target for therapies designed to mitigate HBV-related diseases. While other regions of HBx also engage in interactions, the IDR stands out due to its promiscuous binding capabilities [17,115]. Understanding the interactions mediated specifically by this disordered region is essential for developing inhibitors to block HBx’s contribution to viral persistence and tumorigenesis.

## 10. Identification of Key Host Proteins Directly Interacting with the HBx IDR

**Figure 3:**
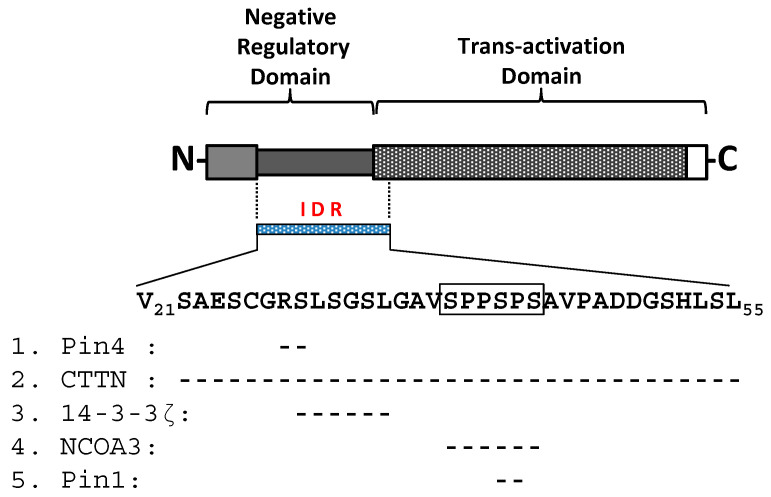
A mapping of host interactors within the intrinsically disordered region (IDR) of HBx. This figure maps five host proteins that specifically interact with the intrinsically disordered region (IDR) of HBx (residues 21–55). The HBx sequence in this region is shown, with key interacting sites indicated with a hyphen sign (--). The five host interactors and their binding regions are as follows: 1. Pin4 (Par14/17 isoforms): interacts with residues 28–29, with an additional site at residues 19–20 (not shown in the figure). 2. Cortactin: binds IDR residues 19–58. 3. 14-3-3ζ protein: interacts with residues 28–33. 4. NCOA3 protein: recognizes the Ser/Pro-rich motif spanning residues 38–43. 5. Pin1 protein: interacts specifically at residues 41–42. The Ser/Pro-rich motif of HBx is highlighted within a box. For the HBx gene, the DNA sequence from GenBank (AIL83994.1) is shown, corresponding to a full-length clone from a Chilean isolate of HBV genotype F1b (KM233681.1).

We conducted an exhaustive review of the literature to first identify a group of host proteins known to directly interact with the intrinsically disordered region of HBx, focusing on cases where specific HBx target sites within the IDR have been mapped. This comprehensive approach allowed us to highlight five key host proteins that play significant roles in HBx-mediated viral replication and oncogenesis: Par14/17, cortactin, 14-3-3ζ, NCOA3, and Pin1 (Figure 3). These interactions are critical as they facilitate the modulation of multiple cellular processes, contributing to HBV pathogenesis and the development of HCC.

### 10.1. Par14/Par17 (PIN4 Gene)

Par14 and Par17, isoforms encoded by the PIN4 gene, belong to the parvulin subfamily of peptidyl–prolyl cis/trans isomerases, which catalyze the isomerization of peptidyl–prolyl bonds [118]. Par14 (13.8 kDa, 131 residues) and Par17 (16.6 kDa, 156 residues) differ by the presence of a 25-residue N-terminal amphipathic α-helix in Par17. Both proteins are implicated in chromatin remodeling, cell cycle control, rRNA processing, and tubulin polymerization [118]. Their expression is elevated in human HCC tissues and HBV-infected cells compared to normal liver cells.

Studies indicate that Par14/Par17 enhance HBV replication by stabilizing HBx, promoting its nuclear and mitochondrial translocation and enhancing cccDNA formation, viral RNA transcription, and virion production [119]. Physical interactions between HBx and Par14/Par17 occur at conserved substrate-binding residues (E46/D74 and E71/D99 in Par14/Par17) and the 19R20P-28R29P motif in HBx, as shown in Figure 3. Their ability to stabilize HBx and promote viral replication highlights their significant role in HBV pathogenesis, making them potential therapeutic targets.

### 10.2. Cortactin (CTTN, 61.6 kDa)

Cortactin is a cytoskeletal protein that regulates actin polymerization and membrane dynamics, playing a key role in processes such as endocytosis, membrane trafficking, and cell migration [120]. It also contributes to cancer progression through its involvement in actin remodeling and lamellipodia formation, which promotes cell migration and invasion. Overexpression of cortactin is common in various cancers, including HCC, and is associated with enhanced metastasis.

HBx interacts with cortactin through its serine/proline-rich motif (residues 19–58), upregulating CREB1 (cAMP response element-binding protein) and downstream targets like cyclin D1 and matrix metalloproteinase-9 (MMP-9), which are critical for cell proliferation and migration [121,122] (Figure 3). This interaction supports tumor progression by suppressing cell cycle arrest. Clinical data show a positive correlation between cortactin and CREB1 expression in HBV-positive HCC tissues, indicating that targeting this interaction could offer a novel therapeutic approach for HCC treatment.

### 10.3. 14-3-3ζ Protein (YWHAZ Gene, 27.7 kDa)

The 14-3-3ζ protein, part of the 14-3-3 protein family, is a highly conserved phospho-serine/threonine-binding protein involved in numerous cellular pathways such as metabolism, apoptosis, and signal transduction [123,124]. Seven isoforms of 14-3-3 exist in mammals, and the ζ isoform is notably overexpressed in HCC tissues.

HBx binds specifically to 14-3-3ζ through its phosphorylated serine residue at position 31 (RPLpS31GP) [125], as indicated in Figure 3. This interaction stabilizes HBx, enhances its activity, and promotes the migratory and invasive capabilities of HCC cells. Inhibiting this interaction leads to increased HBx degradation and reduced cell migration and invasion. Overexpression of both 14-3-3ζ and HBx is positively correlated in HCC tissues, suggesting that disrupting their interaction could be an effective strategy for inhibiting HBV-related tumor progression [125].

### 10.4. NCOA3 (Nuclear Receptor Coactivator 3, 155 kDa)

NCOA3 (also known as amplified in breast cancer 1, AIB1) is a transcriptional coactivator that enhances the activity of nuclear hormone receptors through histone acetyltransferase activity. It is highly expressed in HCC and promotes cancer progression by driving cell proliferation and invasiveness [126,127].

HBx interacts with NCOA3 via a serine/proline motif (38-SSPSPS-43), preventing its degradation by inhibiting ubiquitination through the F-box/WD repeat-containing protein 7 (Fbw7) ubiquitin ligase, a tumor suppressor [128,129], as shown in Figure 3. The stabilization of NCOA3 by HBx promotes the activation of NF-κB signaling and the expression of MMP-9, a key factor in tumor invasion. Mutant forms of HBx that cannot bind NCOA3 exhibit reduced oncogenic activity, underscoring the importance of this interaction in HBV-associated HCC.

### 10.5. Pin1 (Peptidyl-prolyl cis/trans Isomerase, 18.2 kDa)

Pin1 catalyzes the isomerization of phosphorylated Ser/Thr-Pro motifs, affecting the conformation and function of its substrates [130,131]. It is frequently overexpressed in HBV-associated HCC and contributes to tumor growth and progression.

HBx interacts with Pin1 via the serine/proline motif (41S-42P), enhancing HBx stability and promoting hepatocarcinogenesis [132,133] (Figure 3). Co-expression of Pin1 and HBx leads to increased cell proliferation and tumor growth, whereas knockdown of Pin1 impairs HBx-induced tumor formation. This interaction highlights Pin1 as a promising therapeutic target for limiting HBV-related cancer progression.

In summary, IDPs and IDRs are key hubs in cellular and viral interaction networks, enabling dynamic and flexible binding essential for biological processes. Their widespread presence, particularly in viruses, supports immune evasion and host manipulation, contributing to pathogenesis. Given their role in diseases like cancer and viral infections, IDRs offer promising therapeutic targets, with future strategies focusing on overcoming their structural flexibility to develop effective treatments.

## 11. Management of Chronic HBV Infection

Chronic hepatitis B is a liver infection that can cause severe complications, including liver cirrhosis and HCC. The main purposes of HBV therapy are to inhibit viral replication and decrease liver inflammation to mitigate disease progression. Several antiviral medications are approved to treat chronic HBV infection [134,135]. These treatments primarily work by suppressing HBV replication and reducing the viral load, though they rarely achieve complete virus elimination.

A major class of HBV treatments includes nucleos(t)ide analogs, which inhibit the activity of HBV DNA polymerase/reverse transcriptase, thereby preventing viral genome replication. While these agents effectively control viral replication, they often do not fully eradicate the circulating virus, even after extended use [134,136]. Some key nucleos(t)ide analogs include (i) entecavir: known for its high genetic barrier to resistance, it is often the first-line option; (ii) tenofovir: effective against both wild-type and drug-resistant strains of HBV and frequently recommended for patients co-infected with HIV; (iii) tenofovir alafenamide: a newer version of tenofovir with improved safety regarding kidney function and bone health; (iv) lamivudine: one of the earlier antivirals for HBV, but its use is now limited due to high rates of drug resistance; (v) adefovir: less commonly used today because of its lower potency and risk of kidney toxicity; (vi) telbivudine: its clinical use is restricted due to its susceptibility to resistance, particularly cross-resistance with lamivudine [134,135,136]. On the other hand, interferon therapy, particularly with pegylated interferon α-2a, represents another treatment modality. It works by stimulating the immune signaling response against the virus infection and is typically administered for over 48 weeks. This treatment is most effective in younger patients with lower viral loads and no signs of cirrhosis. In advanced cases of liver damage, such as decompensated cirrhosis or liver cancer, a liver transplant may be required. Post-transplant, lifelong antiviral therapy is necessary to prevent the recurrence of HBV infection in the new liver [134,137].

As we already described, chronic HBV remains a major global health burden, with approximately 257 million people affected worldwide. It is a leading cause of liver disease, including cirrhosis, liver failure, and HCC, which result in significant morbidity and mortality. Unfortunately, as indicated, current antiviral treatments are associated with several limitations, including their inability to fully eradicate the virus [10].

## 12. Persistence of HBV and Challenges of Current Therapies

Despite the effectiveness of current antiviral therapies, including nucleos(t)ide analogs and interferon, achieving a complete cure for chronic hepatitis B remains a major challenge [138,139]. These treatments typically suppress HBV replication and reduce viral markers but do not fully eradicate the virus. Prolonged use of these therapies often results in the development of drug-resistant viral strains and adverse side effects, which can compromise patient adherence and quality of life [139].

A key obstacle to HBV eradication is the persistence of the cccDNA within hepatocytes, which serves as a stable reservoir for the virus. This reservoir enables long-term infection and potential reactivation, even after apparent viral suppression [86,140,141]. Thus, targeting or permanently silencing cccDNA is a critical step toward achieving a functional or complete cure. This goal requires innovative antiviral strategies targeting multiple stages of the HBV life cycle.

Recent advances in understanding HBV’s biology, particularly its interactions with host proteins such as the HBx protein, offer promising avenues for the development of next-generation antiviral therapies. HBx plays a key role in regulating cccDNA transcription, viral replication, and immune evasion, making it an ideal target for novel treatments. Although HBx has historically been underexplored as a direct antiviral target, several emerging strategies are currently being evaluated in preclinical and clinical settings. These approaches generally fall into three categories: (i) accelerating HBx protein degradation, primarily through small molecules that trigger proteasome-mediated breakdown; (ii) suppressing HBx gene expression by employing antisense technologies, RNA-targeting agents, or transcriptional inhibitors; and (iii) the development of immunotherapeutic strategies, such as therapeutic vaccines, designed to elicit immune responses specifically against HBx-expressing cells [142,143]. Concurrently addressing these challenges through targeted drug development can help achieve the WHO’s objective of eliminating viral hepatitis as a global public health threat by 2030.

## 13. Targeting the HBx IDR and Interaction Partners with Direct-Acting Antiviral Agents (DAAs)

The success of direct-acting antiviral agents (DAAs) in treating chronic viral infections, particularly HCV infections, underscores their potential application in HBV therapy [144]. HCV is a human, positive-sense, single-stranded RNA virus with a genome of approximately 9.6 kb [145]. It belongs to the *Flaviviridae* family and is classified as a hepacivirus, primarily infecting human liver cells (hepatocytes) [145]. HCV is transmitted through parenteral, sexual, and vertical routes. Among HCV-infected individuals, approximately 60–85% develop a chronic infection if left untreated [146]. The virus encodes a single open reading frame that is translated into a large polyprotein. Within infected cells, this polyprotein is subsequently cleaved by both host and viral proteases into at least ten functional viral proteins [145]. These proteins are categorized as structural proteins (Core, E1, E2, and p7), which form viral particles, and non-structural components like proteases (NS2, NS3, NS4A) responsible for processing the viral polyprotein, as well as the RNA replication machinery components NS4B, NS5A, and NS5B (the viral RNA-dependent RNA polymerase) [111].

DAAs function by directly targeting the key viral components essential for replication, bypassing the need for host immune activation, as seen with traditional therapies like interferon [147]. In the case of HBV, the IDR of the HBx protein, spanning residues 21–55, presents an especially promising target for DAAs (Figure 1A–C) [17]. This region serves as a hub for interactions with multiple host proteins, including 14-3-3ζ, Par14/17, cortactin, Pin1, and NCOA3 (Figure 3). These interactions are critical for HBV replication, viral persistence, and oncogenesis. By disrupting these protein–protein interactions, DAAs targeting the HBx IDR could effectively block key processes driving viral pathogenesis and the development of HCC [147].

Notably, in the case of HCV, DAA therapy does not target the viral entry pathway but instead focuses on inhibiting replication within cells that are already infected [111,147]. This approach focuses on the suppression of active viral replication and the disruption of the viral life cycle, a strategy that is highly relevant for addressing chronic HBV infections [147]. Importantly, the number of functional viral proteins expressed is not a primary concern if the DAA molecule is capable of efficiently targeting and inhibiting a critical viral protein or function. By specifically targeting the HBx IDR, DAAs could neutralize essential pathways sustaining HBV replication and persistence, offering a promising complementary approach to existing antiviral therapies.

## 14. The Broader Potential of DAAs in HBV Treatment Results

The extraordinary success of direct-acting antivirals in treating HCV provides a compelling model for HBV therapy [147]. Drugs directly targeting viral proteins, such as sofosbuvir (a nucleotide analog prodrug directed against the viral RNA polymerase NS5B), ledipasvir and velpatasvir (inhibitors of NS5A, a protein involved in the formation of the RNA HCV replication complex), and glecaprevir (inhibitor of NS3/4a protease), have demonstrated cure rates exceeding 95%, significantly improving patient outcomes [147,148,149]. Unlike older interferon-based regimens, DAAs for HCV are highly effective, require shorter treatment durations (8–12 weeks), and are associated with fewer side effects. Similarly, HBx-targeting DAAs could complement existing HBV therapies by addressing the limitations of current treatments, including the inability to eliminate cccDNA or prevent long-term viral persistence. On the other hand, DAAs targeting HBx, particularly its IDR and associated interactions, would offer several advantages: (i) Targeted cccDNA regulation: by inhibiting HBx-mediated activation of cccDNA transcription, DAAs could reduce viral replication at its basis, addressing the fundamental issue of viral persistence. (ii) Reduced resistance development: targeting multiple host–protein interactions within the HBx IDR could reduce the risk of resistance, as viruses typically struggle to adapt to multi-faceted disruptions. (iii) Oncogenesis suppression: since the HBx IDR plays a crucial role in promoting HCC through interactions with NCOA3, cortactin, and other factors (Figure 3), inhibiting these interactions would simultaneously reduce both viral replication and cancer progression.

## 15. The Future of HBV Therapy Through HBx-Directed DAAs

In summary, the persistence of HBV infection and the associated risk of HCC necessitate innovative therapeutic solutions beyond existing antiviral regimens. The development of direct-acting antiviral agents targeting the IDR of the HBx protein presents a promising avenue for addressing the key challenges of HBV treatment. By interfering with critical HBx–host interactions, these DAAs could suppress viral replication, reduce or abolish cccDNA activity, and mitigate oncogenic signaling pathways. As research progresses, combining HBx-targeting DAAs with existing nucleos(t)ide analogs could lead to synergistic effects, enhancing the likelihood of achieving a functional cure. Furthermore, targeting the HBx IDR and its interaction partners (Figure 3) could offer dual benefits: halting HBV replication while preventing or reducing HCC development. The potential for HBx-directed DAAs to work across different HBV genotypes and stages of infection makes them an engaging candidate for global implementation. Continued research and clinical development in this area will be crucial for overcoming the limitations of current therapies and meeting the WHO’s ambitious target of eliminating hepatitis B as a major global health concern by 2030. Ultimately, a comprehensive therapeutic strategy involving HBx-targeting DAAs could significantly improve outcomes for millions of people living with chronic HBV infection worldwide, offering renewed hope for long-term disease control and prevention.

## Data Availability

Not applicable.
